# Prevalence of Weight Related Comorbidities and Eligibility for Tirzepatide in an Acute Adult Psychiatric Inpatient Population Prescribed Antipsychotics in Northern Ireland

**DOI:** 10.1192/bjo.2026.11803

**Published:** 2026-06-30

**Authors:** Emma Norris, Aidan Turkington

**Affiliations:** Belfast Health and Social Care Trust, Belfast, United Kingdom

## Abstract

**Aims:**

The National Institute for Health and Care Excellence (NICE) has approved Tirzepatide, a glucagon-like peptide-1 (GLP-1) receptor agonist, for obesity management inadults with Body Mass Index (BMI) ≥35 and one of the following weight-related comorbidities: Type 2 Diabetes Mellitus; hypertension; dyslipidaemia; cardiovascular disease and/or obstructive sleep apnoea. Antipsychotic medications are known to be associated with weight gain and an elevated risk of cardiometabolic disorders. The aim of this audit was to determine prevalence of weight related morbidity and eligibility for Tirzepatide in an acute psychiatric inpatient population prescribed antipsychotics in Northern Ireland.

**Methods:**

Inpatients in one acute adult psychiatric hospital prescribed at least one antipsychotic medication were audited. Demographics, antipsychotic use, BMI, and weight-related comorbidities were extracted. Frequencies and proportions of weight-related comorbidities and of patients eligible for Tirzepatide are presented. Group comparisons were made by sex and antipsychotic type. Statistical significance was performed using chi-squared tests.

**Results:**

Seventy-five patients were audited, 41 females and 34 males. The mean age of the cohort was 46.1 years. Fourteen (18.7%) had a BMI ≥35 and thirty-one patients (41.3%) had at least one weight-related comorbidity. Eleven patients (14.7%) met both criteria for Tirzepatide eligibility.

13 different antipsychotics were prescribed across the 75 patients. The most common antipsychotics used included Olanzapine (15), Aripiprazole (15), Quetiapine (11), Amisulpride (9), Risperidone (9) and Clozapine (9).

Weight-related comorbidities were more prevalent in those prescribed Olanzapine (66.7%, 10/15) compared to other antipsychotics (35.0%, 21/60 (χ²=4.96, p<0.05)).

Of the patients prescribed only a single second-generation antipsychotic 20.4% (10/49) met NICE criteria for Tirzepatide compared to 0% (0/10) of patients prescribed a single first-generation antipsychotic (χ²=2.49, p=0.11) and 9.1% (1/11) on aripiprazole only (χ²=0.95, p=0.33).

**Conclusion:**

Weight-related comorbidities were more prevalent in patients prescribed Olanzapine compared to other antipsychotics. Up to 14% of this acute psychiatric inpatient population prescribed antipsychotics in Northern Ireland may be eligible for Tirzepatide for management of obesity.

